# *Aggregatibacter aphrophilus* pacemaker endocarditis: a case report

**DOI:** 10.1186/1756-0500-7-885

**Published:** 2014-12-08

**Authors:** Sahil R Patel, Nishi H Patel, Amit Borah, Heath Saltzman

**Affiliations:** Department of Internal Medicine, Drexel University College of Medicine, Philadelphia, Pennsylvania USA; Department of Pulmonary/Critical Care, Drexel University College of Medicine, Philadelphia, Pennsylvania USA; Department of Cardiology, Drexel University College of Medicine, Philadelphia, Pennsylvania USA

**Keywords:** Endocarditis, HACEK, Pacemaker, Aggregatibacter, Haemophilus, CIED

## Abstract

**Background:**

*Aggregatibacter* bacteria are a rare cause of endocarditis in adults. They are part of a group of organisms known as HACEK – *Haemophilus*, *Aggregatibacter*, *Cardiobacter*, *Eikenella*, and *Kingella*. Among these organisms, several *Haemophilus* species have been reclassified under the genus *Aggregatibacter*. Very few cases of *Aggregatibacter* endocarditis in patients with pacemaker devices have been reported.

**Case presentation:**

We present here what we believe to be the first case of *Aggregatibacter aphrophilus* pacemaker endocarditis. A 62-year-old African American male with a medical history significant for dual-chamber pacemaker placement in 1996 for complete heart block with subsequent lead manipulation in 2007, presented to his primary care doctor with fever, chills, night sweats, fatigue, and ten-pound weight loss over a four-month period. Physical examination revealed a new murmur and jugular venous distension which prompted initiation of antibiotics for suspicion of endocarditis. Both sets of initial blood cultures were positive for *A. aphrophilus*. Transesophageal echocardiogram revealed vegetations on the tricuspid valve and the right ventricular pacemaker lead (Figure 1). This case highlights the importance of identifying rare causes of endocarditis and recognizing that treatment may not differ from the standard treatment for typical presentations. The patient received intravenous ceftriaxone for his endocarditis for a total of six weeks. Upon device removal, temporary jugular venous pacing wires were placed. After two weeks of antibiotic treatment and no clinical deterioration, a new permanent pacemaker was placed and the patient was discharged home.

**Conclusions:**

This is the first case of *A. aphrophilus* endocarditis in a patient with a permanent pacemaker. Our patient had no obvious risk factors other than poor dentition and a history of repeated pacemaker lead manipulation. This suggests that valvulopathies secondary to repeated lead manipulation can be clinically significant factors in morbidity and mortality in this patient population.

## Background

Pacemaker endocarditis caused by *Aggregatibacter* species is rarely described. We present a case of pacemaker endocarditis secondary to *Aggregatibacter aphrophilus*, an organism not previously described in the literature as causing pacemaker endocarditis. *Aggregatibacter* species are a member of the HACEK group of organisms, which also include *Haemophilus*, *Cardiobacter*, *Eikenella corrodens*, and *Kingella*. HACEK organisms are part of the normal oropharyngeal flora, and typically account for 1-3% of native valve endocarditis [1, 2]. In recent years, the number of cardiac devices implanted in patients has increased. Unusually, the number of cardiovascular implantable electronic device (CIED) infections has risen by a greater proportion [3, 4]. Endocarditis has been described in up to 7-10% of cases of pacemaker-related infections [3–6]. Typically, *Staphylococci* are the primary organism responsible for CIED endocarditis [4]. Several large retrospective reviews have not reported HACEK endocarditis in patients with CIEDs [1, 2], although there have been case reports published [7–9].

## Case presentation

The patient is a 62 year old African American male with a medical history significant for complete heart block treated with dual chamber pacemaker implantation in 1996. This was revised in 2007 secondary to a ventricular lead fracture with total left subclavian vein occlusion necessitating lead placement in the right subclavian vein. He initially presented to his primary care physician with a four month history of subjective fevers, chills, night sweats, vomiting, fatigue, decreased energy, and a ten pound weight loss. Physical exam in the office revealed a new systolic murmur consistent with tricuspid regurgitation (TR) and jugular venous distention (JVD). Blood cultures were drawn as an outpatient. One week later, blood cultures were positive for *Aggregatibacter aphrophilus*, and the patient was told to go to the Emergency Department (ED). In the ED the patient was afebrile, with a heart rate of 70 beats/minute and a blood pressure of 130/76 mmHg. His pulse oximetry was 96% on room air. There was no erythema or tenderness over the pacemaker pocket. A TR murmur and JVD were again noted. No lesions were noted on the skin. Initial laboratory values showed a white blood cell count of 14,200 with a differential of 75% neutrophils, 17% lymphocytes, and 1% monocytes. The erythrocyte sedimentation rate was 91 mm/hour. A urinalysis was negative. Peripheral blood cultures were also drawn; subsequently the patient was given a dose of 3.375 g intravenous (IV) piperacillin/tazobactam by the ED physician.

**Figure 1:**
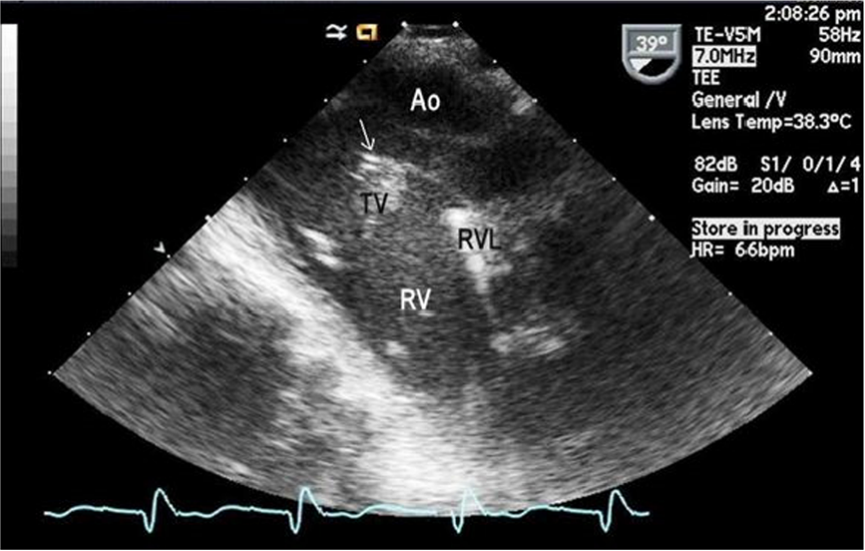
**Transesophageal echocardiogram showing a 1.2 × 0.7 cm vegetation (arrow) attached to the right ventricular pacemaker lead in the right atrium at the coaptation of the tricuspid valve leaflets.** Ao = Aorta, TV = Tricuspid Valve, RVL = Right Ventricular Lead, RV = Right Ventricle.

The patient was admitted to the general medical floor with a high clinical suspicion of HACEK endocarditis and antibiotics changed to ceftriaxone IV 2 g Q24 hours based on outpatient culture susceptibilities that showed beta lactamase negative strains susceptible to penicillin, ampicillin, amoxicillin, gentamycin, ciprofloxacin, and ceftriaxone. On the night of admission, the patient was febrile with a temperature of 101 degrees F (38.3 C). A stat transesophageal echocardiogram (TEE) revealed a 1.2 cm × 0.7 cm vegetation attached to the posterior leaflet of the tricuspid valve and a smaller vegetation attached to the right ventricular pacemaker lead near the coaptation of the tricuspid valve leaflets (Figure 1). He underwent a pacemaker extraction with removal of his device and all leads. Intraoperatively, lead sites and the pocket were debrided with an antibiotic solution. Tissue and extracted leads were also swabbed and cultured. Following extraction, a temporary jugular pacer was placed and the patient was transferred to the Cardiac Care Unit. On the fifth day after admission cultures confirmed beta lactamase negative *Aggregatibacter aphrophilus* with the same susceptibilities reported as outpatient blood cultures.

Following lead extraction, the patient remained afebrile and clinically stable. Repeated blood cultures were persistently negative. Echocardiography prior to new device placement showed residual casts on the tricuspid valve and the junction of superior vena cava and right atrium. A new dual chamber permanent pacemaker was then surgically deployed. He remained stable and was discharged five days later with a Hickman® catheter to finish the remaining 28 days of ceftriaxone for a total of 42 days of antibiotics.

## Discussion

*Aggregatibacter aphrophilus,* first described in 1940 by Khairat and colleagues (initially as *Haemophilus aphrophilus*) is a member of the group of HACEK organisms [10]. Notably, *Aggregatibacter aphrophilus* now includes species formerly known as *H. paraphrophilus* and *H. aphrophilus* [11].

As discussed earlier HACEK organisms account for a small percentage of cases of endocarditis. The occurrence of HACEK organisms in CIED patients is limited to case reports in the current literature. We believe that our case is the first reported case of *Aggregatibacter aphrophilus* endocarditis in a pacemaker-dependent patient. Typically *Aggregatibacter aphrophilus* is part of normal oral flora and frequently found in dental plaques and gingival scrapings [11, 12]. Dental procedures, tongue piercings, use of tongue scrapers, and recent upper respiratory tract infections are known causes for bacterial entry into the bloodstream [9, 11]. In our case, the patient did not report any of the above mentioned factors. However, his exam was significant for markedly poor dentition.

We theorize that the patient may have seeded the bacteria while brushing his teeth. It is well known that this activity increases the risk of transient bacteremia several-fold [12, 13], and given his lack of any other risk factors, this seemed most plausible. Additionally, it is thought that *A. aphrophilus* is similar to *Streptococci* species in that it has a tendency to be virulent only in a predisposed, structurally defective heart [12, 14]. The presence of pacemaker leads is known to cause tricuspid valvulopathy [15]. It is likely that our patient’s repeated lead manipulations caused a valvulopathy which served as a nidus for bacterial seeding.

Our treatment method of *A. aphrophilus* pacemaker endocarditis was similar to the treatment of pacemaker endocarditis caused by other organisms. Upon device removal, the patient was placed on temporary jugular venous pacing. It was mentioned earlier that temporary pacing is associated with increased risk for future infection; however it was necessary in our patient given a history of complete heart block. Additionally, the patient received antibiotic treatment with ceftriaxone for a total of six weeks in accordance with resulted susceptibilities and published guidelines [16]. After two weeks of antibiotic treatment and no clinical deterioration, a new permanent pacemaker was placed and the patient was discharged home to finish 4 more weeks of IV antibiotics

## Conclusions

After a thorough literature search, we did not find any other cases of *A. aphrophilus* endocarditis in a patient with a permanent pacemaker. In addition, this organism is a rare cause of endocarditis in the general population. Our patient had no obvious risk factors other than poor dentition and a history of repeated pacemaker lead manipulation. This suggests that valvulopathies secondary to repeated lead manipulation can be clinically significant factors in morbidity and mortality in this patient population.

## Consent

Written informed consent was obtained from the patient for publication of this Case Report and any accompanying images. A copy of the written consent is available for review by the Editor-in-Chief of this journal.

## Abbreviations

HACEK: *Haemophilus*, *Aggregatibacter*, *Cardiobacter*, *Eikenella*, *Kingella*
CIED: Cardiovascular implantable electronic device
TR: Tricuspid regurgitation
JVD: Jugular venous distention
ED: Emergency department
IV: Intravenous
TEE: Transesophageal echocardiogram
Ao: Aorta
RV: Right ventricle
RVL: Right ventricular lead
TV: Tricuspid valve.
